# Varying Responses of Vegetation Greenness to the Diurnal Warming across the Global

**DOI:** 10.3390/plants11192648

**Published:** 2022-10-08

**Authors:** Jie Zhao, Kunlun Xiang, Zhitao Wu, Ziqiang Du

**Affiliations:** 1 Institute of Loess Plateau, Shanxi University, Taiyuan 030006, China; 2 College of Natural Resources and Environment, Northwest A & F University, Xianyang 712100, China; 3 Guangdong Ecological Meteorology Center, Guangzhou 510275, China

**Keywords:** varying response, diurnal warming, vegetation activity, NDVI

## Abstract

The distribution of global warming has been varying both diurnally and seasonally. Little is known about the spatiotemporal variations in the relationships between vegetation greenness and day- and night-time warming during the last decades. We investigated the global inter- and intra-annual responses of vegetation greenness to the diurnal asymmetric warming during the period of 1982–2015, using the normalized different vegetation index (NDVI, a robust proxy for vegetation greenness) obtained from the NOAA/AVHRR NDVI GIMMS3g dataset and the monthly average daily maximum (T_max_) and minimum temperature (T_min_) obtained from the gridded Climate Research Unit, University of East Anglia. Several findings were obtained: (1) The strength of the relationship between vegetation greenness and the diurnal temperature varied on inter-annual and seasonal timescales, indicating generally weakening warming effects on the vegetation activity across the global. (2) The decline in vegetation response to T_max_ occurred mainly in the mid-latitudes of the world and in the high latitudes of the northern hemisphere, whereas the decline in the vegetation response to T_min_ primarily concentrated in low latitudes. The percentage of areas with a significantly negative trend in the partial correlation coefficient between vegetation greenness and diurnal temperature was greater than that of the areas showing the significant positive trend. (3) The trends in the correlation between vegetation greenness and diurnal warming showed a complex spatial pattern: the majority of the study areas had undergone a significant declining strength in the vegetation greenness response to T_max_ in all seasons and to T_min_ in seasons except autumn. These findings are expected to have important implications for studying the diurnal asymmetry warming and its effect on the terrestrial ecosystem.

## 1. Introduction

Surface vegetation cover is a core component of terrestrial ecosystems, as it plays an important role in connecting material circulation and energy flow in the atmosphere, hydrosphere, and soil circles [1,2,3,4]. Current satellite observations have revealed long-term greening and browning changes in the vegetation greenness (i.e., the normalized difference vegetation index, NDVI) across the global from the 1980s until the present time [2,5,6,7,8]. Such variations in the vegetation activity are closely related to climate change, particularly the increasing global temperatures over the last several decades [1,8,9,10,11], which can be characterized by the increase in both daytime and night-time temperature [12,13]. The link between global warming and vegetation activity is overwhelming. Recent studies have investigated the responses of satellite-derived vegetation productivity to temperatures during daytime and nighttime between regions and ecosystem [12,14,15,16,17,18,19,20]. However, most of the measurements were static and insufficient to clarify the varying responses of vegetation dynamics to climate warming [21].

The relationship between vegetation activity and temperature variations is not fixed; it constantly changes over time because of the interventions of other environmental and anthropogenic factors, in the face of persistent global warming [22,23]. Therefore, the current focus is on the dynamic responses of vegetation greenness to temperature change over time. For instance, Andreu-Hayles et al. [24] explored the varying boreal forest responses to arctic environmental change near the Firth River, Alaska. Piao et al. [23] found a weakening relationship between inter-annual temperature variability and northern vegetation activity. Fu et al. [25] reported declining global warming effects on the phenology of spring leaf unfolding. Cong et al. [26] examined the varied responses of vegetation activity to climate change on the grasslands of the Tibetan Plateau. He et al. [21] identified obvious shifts in the relationship between vegetation growth and temperature in China’s temperate desert and rainforest areas. Although these findings confirm the dynamic relationship between vegetation greenness and temperature change, the link between diurnal warming and vegetation activity, particularly the variations in their correlation over time, remains unclear.

The main objective of this study was to explore the varying relationships between vegetation greenness and day- and night-time warming on temporal and spatial scale, by simultaneously employing time-series satellite-derived vegetation NDVI data and gridded meteorological data. The understandings drawn from the findings are expected to have important implications for studying the diurnal asymmetry warming and its effect on the terrestrial ecosystem.

## 2. Results

### 2.1. Trends of Correlations between Vegetation Greenness and Diurnal Warming

#### 2.1.1. Inter-Annual Changes in R_NDVI-Tmax_ and R_NDVI-Tmin_

**Figure 1:**
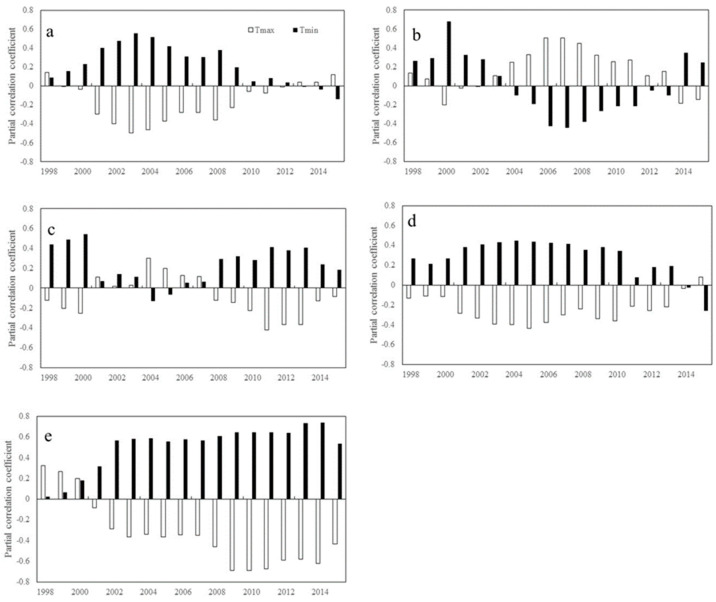
Temporal variations in the partial correlation coefficients between mean annual NDVI and the diurnal temperature (T_max_ and T_min_) for each 17-year moving window across latitudes intervals. (**a**–**e** represents latitudes intervals at 60°~90° N, 30°~60° N, 0°~30° N, −30°~0° S, and −60°~0° S, respectively (N and S indicates the Northern and southern hemisphere, respectively). The x axis is the last year of the 17-year moving-window (for example, 1998 stands for a moving-window from 1982 to 1998, …, 2015 stands for a moving-window from 1999 to 2015). The Y axis is the partial correlation coefficients).

Recent changes in satellite-based vegetation greenness across different areas were closely related to global warming. Figure 1 shows the inter-annual variation in the partial correlation coefficients of the NDVI and diurnal temperature for the period of 1982–2015. In the mid-latitudes of the southern hemisphere, R_NDVI-Tmax_ is approximately 0.323 for the first window of 1982–1998 and decreased to −0.436 for the last window of 1999–2015. In the high latitudes of the northern hemisphere, R_NDVI-Tmin_ shows a significant increasing trend (β = 0.098, *p* < 0.01) in the first six windows and then a significant decreasing trend (β = −0.056, *p* < 0.01) in the remaining windows. Similarly, in the low latitudes of the southern hemisphere, R_NDVI-Tmin_ shows a significant increasing trend (β = 0.039, *p* < 0.01) in the first seven windows, followed by a significant decreasing trend (β = −0.055, *p* < 0.01) in the remaining windows. In contrast, in the mid-latitudes of the southern hemisphere, R_NDVI-Tmin_ is generally on an upward trend, increasing from 0.022 in the window of 1982–1998 to 0.737 in the window of 1998–2014. These findings indicate a notable shift in the responses of vegetation activity to diurnal temperatures over the last three decades.

Overall, R_NDVI-Tmax_ exhibits only a significant downward trend in the mid-latitudes of the southern hemisphere (β = −0.051, *p* < 0.05). In contrast, R_NDVI-Tmin_ shows a significant upward trend in the mid-latitudes of the southern hemisphere (β = 0.032, *p* < 0.05), but a significant downward trend in the low latitudes of the southern hemisphere (β = −0.021, *p* < 0.05). R_NDVI-Tmax_ does not exhibit a significant trend in the northern hemisphere (*p* > 0.05), and R_NDVI-Tmin_ exhibits a significant downward trend only in the high latitudes of the northern hemisphere (β = −0.021, *p* < 0.05).

#### 2.1.2. Inter-Annual Changes in R_NDVI-Tmax_ and R_NDVI-Tmin_

Variations in the temperature on a seasonal scale may not correspond to similar changes on inter-annual scale. The photosynthesis of vegetation in different growth stages depends on the seasonal cycle of the temperature. Therefore, the sensitivity of vegetation greenness to day- and nighttime warming and variations in seasonality is likely to be highly complex. 

**Figure 2:**
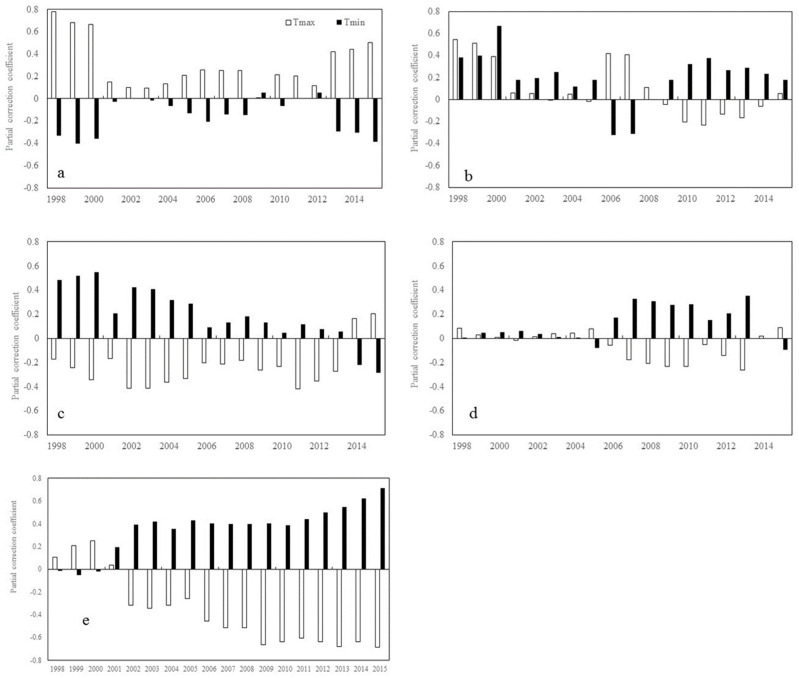
Temporal variations in the partial correlation coefficients between mean NDVI and the diurnal temperature (T_max_ and T_min_) in spring for each 17-year moving window across latitudes intervals. (**a**–**e** represents latitudes intervals at 60°~90° N, 30°~60° N, 0°~30° N, −30°~0° S, and −60°~0° S, respectively (N and S indicates the Northern and southern hemisphere, respectively). The x axis is the last year of the 17-year moving-window (for example, 1998 stands for a moving-window from 1982 to 1998,…, 2015 stands for a moving-window from 1999 to 2015). The Y axis is the partial correlation coefficients).

During spring, R_NDVI-Tmax_ shows a significant downward trend at the mid-latitudes in both the northern hemisphere (β = −0.031, *p* < 0.05) and the southern hemisphere (β = −0.055, *p* < 0.05) (Figure 2). R_NDVI-Tmin_ shows a significant downward trend (β = −0.040, *p* < 0.05) at the low latitudes in the northern hemisphere while a significant upward trend (β = 0.035, *p* < 0.05) at the mid-latitudes in the southern hemisphere (Figure 2).

**Figure 3:**
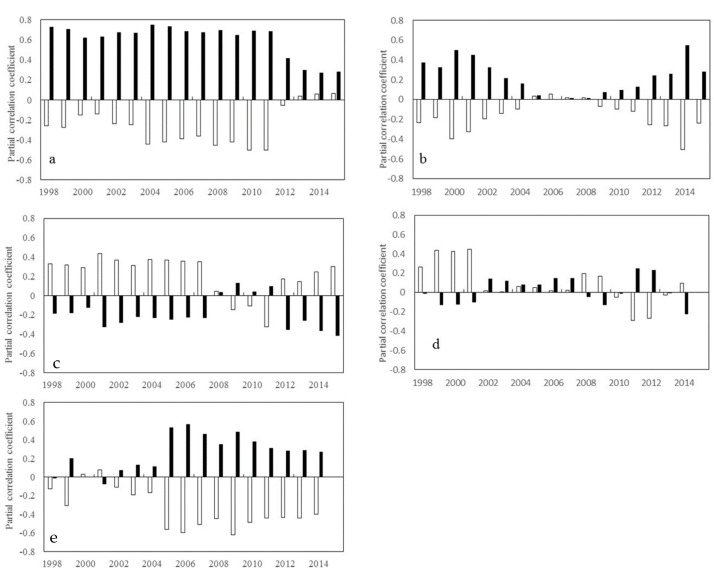
Temporal variations in the partial correlation coefficients between mean NDVI and the diurnal temperature (T_max_ and T_min_) in summer for each 17-year moving window across latitudes intervals. (**a**–**e** represents latitudes intervals at 60°~90° N, 30°~60° N, 0°~30° N, −30°~0° S, and −60°~0° S, respectively (N and S indicates the Northern and southern hemisphere, respectively). The x axis is the last year of the 17-year moving-window (for example, 1998 stands for a moving-window from 1982 to 1998,…, 2015 stands for a moving-window from 1999 to 2015). The Y axis is the partial correlation coefficients).

During summer, R_NDVI-Tmax_ shows a significant downward trend not only at the low latitudes in the northern hemisphere (β = −0.020, *p* < 0.05), but also at the low and mid-latitudes in the southern hemisphere (β = −0.029, *p* < 0.05) (Figure 3). R_NDVI-Tmin_ shows a significant downward trend (β = −0.022, *p* < 0.05) at the high latitudes in the northern hemisphere, but a significant upward trend (β = 0.023, *p* < 0.05) at the mid-latitudes in the southern hemisphere (Figure 3).

**Figure 4:**
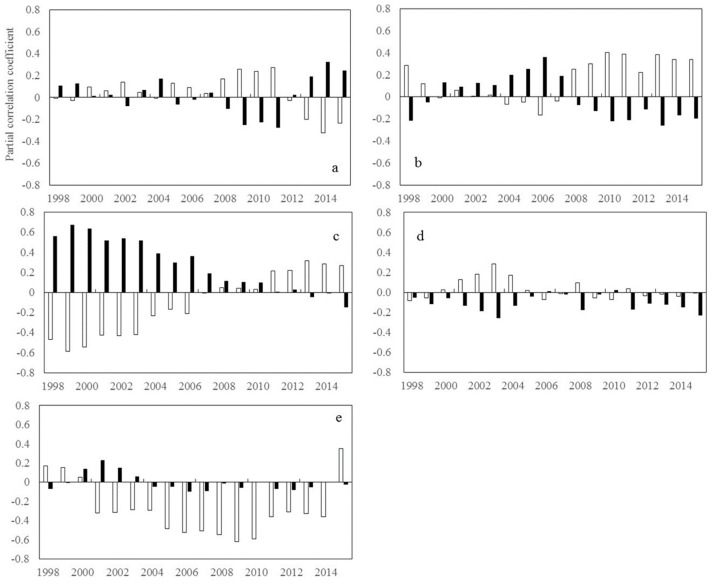
Temporal variations in the partial correlation coefficients between mean NDVI and the diurnal temperature (T_max_ and T_min_) in autumn for each 17-year moving window across latitudes intervals. (**a**–**e** represents latitudes intervals at 60°~90° N, 30°~60° N, 0°~30° N, −30°~0° S, and −60°~0° S, respectively (N and S indicates the Northern and southern hemisphere, respectively). The x axis is the last year of the 17-year moving-window (for example, 1998 stands for a moving-window from 1982 to 1998,…, 2015 stands for a moving-window from 1999 to 2015). The Y axis is the partial correlation coefficients).

During autumn, R_NDVI-Tmax_ shows a significant upward trend at the low-latitudes (β = 0.057, *p* < 0.01) and the mid-latitudes (β = 0.021, *p* < 0.01) in the northern hemisphere. (Figure 4) R_NDVI-Tmin_ shows a significant downward trend at the mid-latitudes (β = −0.017, *p* < 0.05) and the low latitudes (β = −0.048, *p* < 0.01) in the northern hemisphere (Figure 4). However, in the southern hemisphere, we did not find any statistically significant variations in R_NDVI-Tmax_ and R_NDVI-Tmin_.

**Figure 5:**
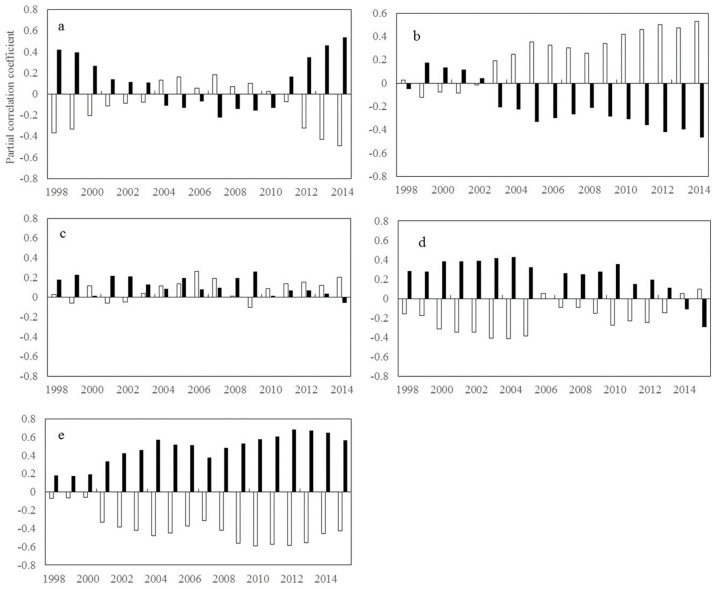
Temporal variations in the partial correlation coefficients between mean NDVI and the diurnal temperature (T_max_ and T_min_) in winter for each 17-year moving window across latitudes intervals. (**a**–**e** represents latitudes intervals at 60°~90° N, 30°~60° N, 0°~30° N, −30°~0° S, and −60°~0° S, respectively (N and S indicates the Northern and southern hemisphere, respectively). The x axis is the last year of the 17-year moving-window (for example, 1998 stands for a moving-window from 1982 to 1998,…, 2015 stands for a moving-window from 1999 to 2015). The Y axis is the partial correlation coefficients).

During winter, R_NDVI-Tmax_ shows a significant upward trend at the mid-latitudes in the northern hemisphere (β = 0.041, *p* < 0.01) and the low latitudes in the southern hemisphere (β = 0.016, *p* < 0.05), but a significant downward trend (β = −0.025, *p* < 0.01) at the mid-latitudes in the southern hemisphere (Figure 5). R_NDVI-Tmin_ shows a significant downward trend at the low (β = −0.010, *p* < 0.05) and the mid-latitudes (β = −0.036, *p* < 0.01) in the northern hemisphere and at the low latitudes (β = −0.025, *p* < 0.01) in the southern hemisphere, but a significant upward trend at the mid-latitudes (β = 0.027, *p* < 0.01) in the southern hemisphere (Figure 5). Although we tried to characterize the sensitivity of vegetation greenness for each season, the possible mechanism leading to the seasonal divergence in the response of vegetation greenness to diurnal warming needs to be studied further.

### 2.2. Spatial Patterns of the Trends in the Correlations between Vegetation Greenness and Diurnal Temperatures

#### 2.2.1. Inter-Annual Patterns of R_NDVI-Tmax_ and R_NDVI-Tmin_

**Figure 6:**
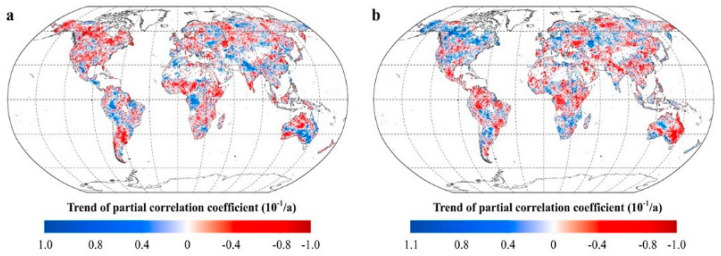
The response of vegetation greenness to the diurnal temperature. (**a**. spatial distribution of the temporal trend of the partial coefficients between mean annual NDVI and T_max_. **b**. spatial distribution of the temporal trend of the partial coefficients between mean annual NDVI and T_min_. Supplementary Table S1).

Figure 6 shows a summary of the spatial variations in the correlations between vegetation activity and diurnal temperatures over 30 years. The areas with a significant increase in R_NDVI-Tmax_ account for 26.01% of the total vegetated areas, mainly located in the southeastern part of Oceania, the Ganges Plain, the western plains of Eastern Europe, and the Amazonian Plain. The areas with a significant decrease in R_NDVI-Tmax_ account for 35.65% of the total vegetated areas, mainly distributed in the northwestern part of Oceania, southern South America, eastern Africa, and the cold temperate zones of the northern hemisphere. About 28.62% of the total vegetated areas show a significant upward trend in R_NDVI-Tmin_, these areas are mainly located in southern South America, southern Africa, Oceania, and the northwestern boreal regions of North America. Over 32.49% of the total vegetated areas shows a significant downward trend in R_NDVI-Tmin_, these areas are mainly distributed in eastern Oceania, central Africa, and the tropical regions in the northern hemisphere. In particular, the number of pixels with a significant decrease in R_NDVI-Tmax_ were greater than those with a significant increase in R_NDVI-Tmax_ in each latitude interval.

This difference in the number of pixels between R_NDVI-Tmax_ and R_NDVI-Tmin_ was most pronounced at the mid-latitudes in the southern hemisphere and the mid- and high latitudes in the northern hemisphere. At the mid- and high latitudes in the northern hemisphere and at the mid-latitudes in the southern hemisphere, the proportion of R_NDVI-Tmin_ showing a significant upward trend is slightly higher than that of R_NDVI-Tmin_ showing a significant downward trend. At low latitudes, however, the percentage of areas where R_NDVI-Tmin_ shows a significant downward trend is much higher than that of areas where R_NDVI-Tmin_ shows the opposite trend.

Overall, regions with a declining correlation between vegetation greenness and diurnal temperatures were more than those with an increasing correlation. This implies that worldwide vegetation activity on a larger scale became less sensitive to variations in the day- and night temperature with global warming during the last several decades.

#### 2.2.2. Intra-Annual Patterns of R_NDVI-Tmax_ and R_NDVI-Tmin_

**Figure 7:**
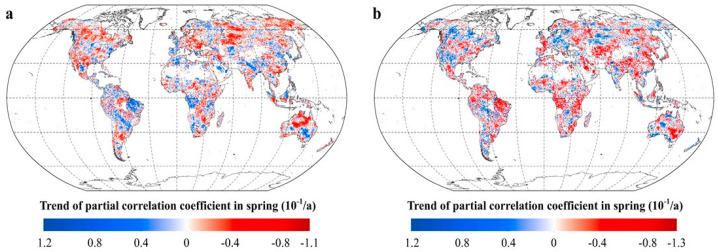
The response of vegetation greenness to the diurnal temperature in spring. (**a**. spatial distribution of the temporal trend of the partial coefficients between spring NDVI and T_max_. **b**. spatial distribution of the temporal trend of the partial coefficients between spring NDVI and T_min_. Supplementary Table S2).

The seasonal variation in the vegetation greenness response to global warming may increase the complexity of the intra-annual patterns of R_NDVI-Tmax_ and R_NDVI-Tmin_. For spring, the areas with positive trends in R_NDVI-Tmax_ accounts for 25.66% of the total vegetated areas, mostly in the northeastern part of South America, the eastern plains of Eastern Europe, the Iberian Peninsula, the southeastern parts of Australia, and the Ganges Plain (Figure 7). The areas with negative trends in R_NDVI-Tmax_ accounts for 32.43% of the total vegetated areas, mainly distributed in Western Australia, the Katanga Plateau, and the cold temperate zones in the northern hemisphere. Similarly, about 24.09% of the vegetated areas show positive trends in R_NDVI-Tmin_, these areas are mainly located in northern North America, southern South America, southern Europe, the Indian Peninsula, and southwestern Australia. Over 32.11% of the vegetated areas show negative trends in R_NDVI-Tmin_, these areas are mainly distributed in northern South America, central Africa, southeastern Australia, and the temperate regions of the northern hemisphere.

**Figure 8:**
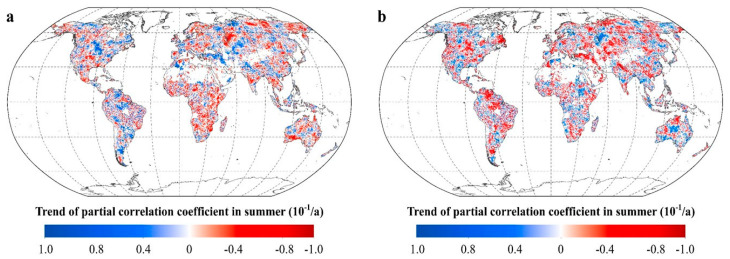
The response of vegetation greenness to the diurnal temperature in summer. (**a**. spatial distribution of the temporal trend of the partial coefficients between summer NDVI and T_max_. **b**. spatial distribution of the temporal trend of the partial coefficients between summer NDVI and T_min_. Supplementary Table S3).

For summer, R_NDVI-Tmax_ shows significant positive trends in 29.80% of the vegetated areas, located mainly in the northern and eastern North America, southern South America, Armenian plateau, Tulan lowland, Kazakh hills, and Ganges plain (Figure 8). R_NDVI-Tmax_ shows significant negative trends in 31.78% of the vegetated areas, located mainly in southern North America, Central European Plains, Scandinavia, Northeast Asia, India Peninsula, and Indochina. Similarly, R_NDVI-Tmin_ shows significant positive trends in 26.68% of the vegetated areas, mainly in the southern Patagonia Plateau of South America, the Yukon Plateau in North America, the south-central United States, the northwestern part of Asia, the Indo-China Peninsula, and Southern Australia. R_NDVI-Tmin_ shows significant negative trends in 34.71% of the vegetated areas, located mainly in northern South America, southern Africa, northwestern Australia, and the cold temperate zones of the northern hemisphere (Figure 8).

**Figure 9:**
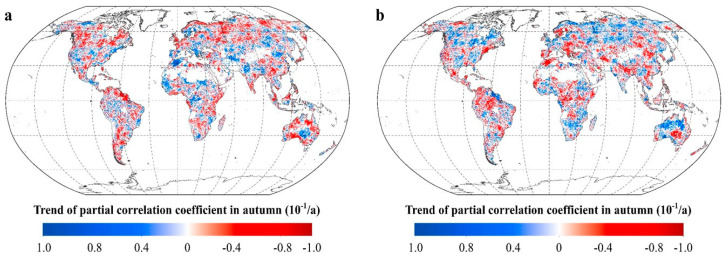
The response of vegetation greenness to the diurnal temperature in autumn. (**a**. spatial distribution of the temporal trend of the partial coefficients between autumn NDVI and T_max_. **b**. spatial distribution of the temporal trend of the partial coefficients between autumn NDVI and T_min_. Supplementary Table S4).

For autumn, about 27.43% of the total vegetated areas show significant positive trends in R_NDVI-Tmax_, these areas are mainly distributed in central Eurasia, central and southern North America, northern Africa, and central Australia (Figure 9). Over 34.07% of the total vegetated areas show significant negative trends in R_NDVI-Tmax_, these areas are mainly distributed in northern Eurasia, central North America, southern Africa, western Africa, southern South America, and northeastern Australia. In contrast, over 32.67% of the total vegetated areas show significant positive trends in R_NDVI-Tmin_, these areas are widely distributed in southern Africa, northern Australia, and the cold temperate zones of the northern hemisphere. About 28.76% of the total vegetated areas show significant negative trends in R_NDVI-Tmin_, these areas are mainly distributed in southern North America, northwestern South America, central Asia, southern Europe, central Africa, and southern Europe (Figure 9).

**Figure 10:**
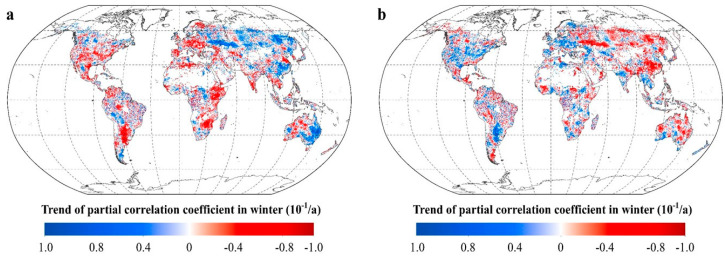
The response of vegetation greenness to the diurnal temperature in winter. (**a**. spatial distribution of the temporal trend of the partial coefficients between winter NDVI and T_max_. **b**. spatial distribution of the temporal trend of the partial coefficients between winter NDVI and T_min_. Supplementary Table S5).

For winter, the positive trends in R_NDVI-Tmax_ are found in 25.06% of the total vegetated areas, located mainly in central Asia, eastern Australia, Eastern Europe, and southern China (Figure 10). The negative trends in R_NDVI-Tmax_ are found in 25.74% of the total vegetated areas, mainly distributed in Western Europe, central and southern North America, central South America, eastern Africa, Western Australia, Indian Peninsula, and Indochina. Similarly, the positive trends in R_NDVI-Tmin_ are found in 23.32% of the total vegetated areas, located mainly in central North America, Western Europe, southwestern Australia, and the north-central La Plata Plain in South America. The negative trends in R_NDVI-Tmin_ are found in 26.71% of the total vegetated areas, mainly distributed in central Asia, central and eastern Australia, and Eastern Europe (Figure 10).

## 3. Discussion

The global climate has undergone continuous warming since the 19th century. Such warming has been characterized by a faster change in the global land surface during the nighttime than during the daytime [12,27,28]. However, the current understanding of the sensitivity of vegetation activity to daytime and nighttime temperatures (not to mean temperature) during the last decades is still poor. Therefore, we applied the moving-window-based partial correlation analysis and ordinary least squares linear regression method within a moving 17-year window to the GIMMS NDVI3g dataset and gridded meteorological data covering the global vegetated areas to reveal the variations in the response of vegetation greenness to the day- and nighttime warming during the period of 1982–2015.

We reported a wide reduction in the vegetation NDVI and diurnal temperature sensitivity since the early 1980s across the global. We also showed that the strength of the relationship between vegetation greenness and diurnal temperature varied among seasons and not just at inter-annual intervals. We further confirmed declining warming effects on vegetation activity, in line with some earlier studies conducted from local to global scale [21,22,23,24,25,26]. 

We tried to reveal the dynamic responses of vegetation greenness to day and night warming and explore the difference in the responses of vegetation to such diurnal warming. However, some uncertainties in this study still constrained our understanding of these responses. For example, the changing response could be related to any one, or a combination, of various factors, such as increasing drought stress [29,30,31], rising CO2 fertilization [22,23], fire disturbances [32,33], global dimming [29], and human-associated activities [21]. These factors were not considered in our analyses because of the lack of sufficient records. Regardless of the cause, our findings on the change in the response of vegetation greenness to diurnal warming have important implications for studies on climate change related to the past as well as the future. Further studies are needed, combining process studies (e.g., ecosystem warming experiments) with multi-scale data, to clarify the mechanisms of the observed decline in the correlation between the diurnal warming and vegetation activity. Our study mainly examined the immediate responses of terrestrial vegetation activity to asymmetric warming. In fact, such asymmetric warming has a non-uniform time-lag impact on terrestrial vegetation growth [34]. Previous studies have also found different responses of vegetation activity to warming across terrestrial ecosystems [12,23]. We did not pay enough attention to this difference in the biome involved. Although the combined effects of hydrothermal regime, ecosystem types, and the time-lag effects increase the uncertainties associated with the weakening relationship between vegetation greenness and current asymmetric warming during day- and night-time, our findings demonstrate the need to consider asymmetric warming and its uneven effect rather than variations in the annual mean temperature when modeling the future behavior of vegetation activity. Hence, significant efforts are required to examine the varying responses of vegetation activity to climate change in such a dynamic climate system in light of the substantial spatial and temporal variations in the diurnal warming and its effects.

## 4. Materials and Methods

### 4.1. Datasets

The third-generation NDVI data for the period of 1982–2015 was produced by the Global Inventory Modelling and Mapping Studies group (GIMMS NDVI3g) from the NOAA/AVHRR series satellite imageries, with a spatial resolution of ~0.083º and fortnightly interval. To further minimize the atmospheric noise, the value composites method was used to reconstruct the monthly and yearly NDVI datasets [35]. These NDVI data were aggregated to 0.5º×0.5º to match the resolution of Meteorological data. Pixels with the mean NDVI (annual and monthly) below 0.1 served as non-vegetation areas and were employed as a mask [5,26]. The GIMMS NDVI3g can be used as a proxy for vegetation greenness and has been widely applied to explore the diverse spatiotemporal-scale vegetation activity [5,12,23,26,34].

Climatological data for the same period, including the monthly average daily maximum (T_max_) and minimum temperature (T_min_) and precipitation, with a spatial resolution of ~ 0.5° × 0.5° were obtained from the gridded Climate Research Unit, University of East Anglia (CRU TS 4.01). The CRU TS 4.01 data were produced using angular-distance weighting interpolation. These meteorological materials have been well used in studies on the relationship between global and regional vegetation cover and climate change [7,12,23,34,36].

### 4.2. Methods

#### 4.2.1. Moving-Window-Based Partial Correlation Analysis

The relationships between vegetation NDVI and temperature were examined using partial correlation analysis (the partial correlation coefficient of NDVI and day- and nighttime temperature were denoted by R_NDVI-Tmax_ and R_NDVI-Tmin_, respectively), to eliminate the covariate effects of other factors [12,37,38,39]. In this case, for instance, the partial correlation coefficient between NDVI and T_max_ was calculated when conditioning the precipitation and T_min_; and that between NDVI and T_min_ was calculated when conditioning the precipitation and T_max_. The partial correlations of the NDVI with temperature were calculated in the first 17-year window (1982–1998). The window was then moved forward by 1 year to represent the second 17-year window (1983–1999), and so on until the last 17-year window (1999–2015). Thus, we obtained the partial correlation coefficients within all 17-year windows, which were applied to describe the responses of vegetation greenness to day- and nighttime warming. The NDVI and relevant variables were detrended by a linear detrending method prior to the partial correlation analysis [12,17].

#### 4.2.2. Ordinary Least Squares Linear Regression 

To explore the dynamics in the correlation between global vegetation greenness and day- and night temperature, the Ordinary least-squares linear regression analysis method was used to calculate the slope of the partial correlation coefficients of NDVI and T_max_ and T_min_ within each 17-year window over the entire temporal domain. The partial correlation coefficient and P-value were used to determine the relationship and its significance. The slope of the linear regression represents the changing trend in the correlations between the vegetation greenness and diurnal warming during the period 1982–2015. A positive slope indicates a positive growth response to temperature, while a negative slope indicates a weakening relationship with diurnal warming.

(1) $$y_{\left(t\right)}=\alpha +\beta t+\varepsilon$$

Here, α and β were the fitted intercept and slope, respectively. A two-tailed *t*-test was used to examine the significance level of the trend. Thus, we described the change in vegetation greenness responses to the diurnal warming from 1982 to 2015. 

## 5. Conclusions

In the most recent period (i.e., 1982–2015), most parts of the world exhibited an overall significant change in the response of vegetation greenness to diurnal warming. On an interannual timescale, the percentage of areas showing a significantly negative trend in the partial correlation coefficient between vegetation greenness and day- and nighttime temperatures was greater than that of areas showing a significant positive trend. For daytime temperatures, this decline in the vegetation response occurred mainly at the mid-latitudes of the world and at the high latitudes of the northern hemisphere. For nighttime temperatures, the decline was primarily concentrated at the low-latitudes across the global. Our study also showed that the strength of the associations between vegetation greenness and diurnal warming varied among seasons. Although the trends in the correlation between vegetation greenness and diurnal warming showed a complex spatial pattern, most of the study areas had undergone a significant declining strength in the vegetation greenness response to daytime temperatures in all seasons and to nighttime temperatures in seasons except autumn. This further implies that the recent reduction in the strength of the response of vegetation greenness to diurnal temperature fluctuations became widespread throughout the world as such as the inter-annual tendency. These findings shew new light on our understanding of the response of vegetation greenness to global warming. We demonstrate the need to carefully consider the asymmetric diurnal warming when unraveling the influence of global warming on terrestrial ecosystem. 

## Data Availability

The data used in the present work have been listed in the Supplementary Materials.

## Supplementary Materials

The following supporting information can be downloaded at: https://www.mdpi.com/article/10.3390/plants11192648/s1, Table S1: Slopes of the partial correlation coefficients between mean annual NDVI and the duirnal temperature (T_max_ and T_min_) for each 17-year moving window across latitudes intervals; Table S2: Slopes of the partial correlation coefficients between mean NDVI and the duirnal temperature (T_max_ and T_min_) in spring for each 17-year moving window across latitudes intervals; Table S3: Slopes of the partial correlation coefficients between mean NDVI and the duirnal temperature (T_max_ and T_min_) in summer for each 17-year moving window across latitudes intervals; Table S4: Slopes of the partial correlation coefficients between mean NDVI and the duirnal temperature (T_max_ and T_min_) in autumn for each 17-year moving window across latitudes intervals; Table S5: Slopes of the partial correlation coefficients between mean NDVI and the duirnal temperature (T_max_ and T_min_) in winter for each 17-year moving window across latitudes intervals.
