# Supplementary material for: Varying Responses of Vegetation Greenness to the Diurnal Warming across the Global
Source: Plants (Basel). 2022 Oct 8;11(19):2648. doi: 10.3390/plants11192648 (PMC9571579; doi:10.3390/plants11192648)
Supplement: Supplementary file 1 [file plants-11-02648-s001.zip › plants-1941239-supplementary.pdf]

Supplementary:

Table S1 Slopes of the partial correlation coefficients between mean annual NDVI and the duirnal temperature (T_{max} and T_{min}) for each 17-year moving window across latitudes intervals. (The slope represents represents the change trend in the correlations between the vegetation greenness and diurnal warming during the period 1982–2015. A positive slope indicates a positive growth response to temperature, while a negative slope indicates a weakening relationship with diurnal warming.p indicates the statistically significance level of the change trend tested by a two-tailed t-test.at the 0.05 level. N and S indicates the Northern and sourthern hemisphere, respectively.)

Latitudes	$R_{NDVI-Tmax}$		$R_{NDVI-Tmin}$	
	Slope	*p*	Slpoe	*p*
60° - 90°N	0.012	0.209	-0.023	0.008
30°- 60°N	0.002	0.861	-0.023	0.124
00°- 30°N	-0.014	0.130	0.001	0.953
-30°- 00°S	0.006	0.351	-0.021	0.012
-60° - -30°S	-0.051	0.000	0.032	0.000

Table S2 Slopes of the partial correlation coefficients between mean NDVI and the duirnal temperature (T_{max} and T_{min}) in spring for each 17-year moving window across latitudes intervals. (The slope represents represents the change trend in the correlations between the vegetation greenness and diurnal warming during the period 1982–2015. A positive slope indicates a positive growth response to temperature, while a negative slope indicates a weakening relationship with diurnal warming.*p* indicates the statistically significance level of the change trend tested by a two-tailed t-test.at the 0.05 level. N and S indicates the Northern and sourthern hemisphere, respectively.)

Latitudes	$R_{NDVI-Tmax}$		$R_{NDVI-Tmin}$	
	Slope	*p*	Slpoe	*p*
60° - 90°N	-0.011	0.305	0.003	0.722
30°- 60°N	-0.031	0.002	-0.008	0.453
00°- 30°N	0.014	0.073	-0.040	0.000
-30°- 00°S	-0.010	0.065	0.009	0.178
-60° - -30°S	-0.055	0.000	0.035	0.000

Table S3 Slopes of the partial correlation coefficients between mean NDVI and the duirnal temperature (T_{max} and T_{min}) in summer for each 17-year moving window across latitudes intervals. (The slope represents represents

the change trend in the correlations between the vegetation greenness and diurnal warming during the period 1982–2015. A positive slope indicates a positive growth response to temperature, while a negative slope indicates a weakening relationship with diurnal warming.p indicates the statistically significance level of the change trend tested by a two-tailed t-test.at the 0.05 level. N and S indicates the Northern and sourthern hemisphere, respectively.)

Latitudes	$R_{NDVI-Tmax}$		$R_{NDVI-Tmin}$	
	Slope	*p*	Slpoe	*p*
60° - 90°N	0.010	0.267	-0.022	0.001
30°- 60°N	-0.001	0.839	-0.007	0.387
00°- 30°N	-0.020	0.037	-0.001	0.917
-30°- 00°S	0.029	0.002	0.004	0.603
-60° - -30°S	-0.029	0.003	0.023	0.014

Table S4 Slopes of the partial correlation coefficients between mean NDVI and the duirnal temperature (T_{max} and T_{min}) in autumn for each 17-year moving window across latitudes intervals. (The slope represents represents the change trend in the correlations between the vegetation greenness and diurnal warming during the period 1982–2015. A positive slope indicates a positive growth response to temperature, while a negative slope indicates a weakening relationship with diurnal warming.p indicates the statistically significance level of the change trend tested by a two-tailed t-test.at the 0.05 level. N and S indicates the Northern and sourthern hemisphere, respectively.)

Latitudes	$R_{NDVI-Tmax}$		$R_{NDVI-Tmin}$	
	Slope	*p*	Slpoe	*p*
60° - 90°N	-0.009	0.213	0.002	0.830
30°- 60°N	0.021	0.009	-0.017	0.043
00°- 30°N	0.057	0.000	-0.048	0.000
-30°- 00°S	-0.005	0.260	-0.002	0.622
-60° - -30°S	-0.083	0.173	-0.007	0.073

Table S5 Slopes of the partial correlation coefficients between mean NDVI and the duirnal temperature (T_{max} and T_{min}) in winter for each 17-year moving window across latitudes intervals. (The slope represents represents the change trend in the correlations between the vegetation greenness and diurnal warming during the period 1982–2015. A positive slope indicates a positive growth response to temperature, while a negative slope indicates a weakening relationship with diurnal warming.p indicates the statistically significance level of the change trend tested by a two-tailed t-test.at the 0.05 level. N and S indicates the Northern and sourthern hemisphere, respectively.)

Latitudes	$R_{NDVI-Tmax}$		$R_{NDVI-Tmin}$	
	Slope	*p*	Slpoe	*p*
60° - 90°N	-0.003	0.787	-0.000	0.992
30°- 60°N	0.041	0.000	-0.036	0.000
00°- 30°N	-0.009	0.069	-0.010	0.026
-30°- 00°S	0.016	0.028	-0.025	0.001
-60° - -30°S	-0.025	0.000	0.027	0.000
